# Enhancing Data Integrity in Online Health Surveys Through Multilayered Security Measures: Cross-Sectional Study

**DOI:** 10.2196/93703

**Published:** 2026-09-02

**Authors:** Maria Mora Pinzon, Susana Fernandez de Cordova, Maichou Lor

**Affiliations:** 1 Department of Medicine, Division of Geriatrics and Gerontology, School of Medicine and Public Health, University of Wisconsin–Madison, 610 Walnut St, Madison, WI, 53726, United States, 1 6088902524; 2 School of Nursing, University of Wisconsin–Madison, Madison, WI, United States

**Keywords:** online surveys, bots, fraud, verification, security measures, data integrity

## Abstract

A multilayered fraud-mitigation approach is essential to ensure data integrity in medical survey research; basic measures alone (eg, CAPTCHA) would permit widespread fraud.

## Introduction

Online health surveys have become essential tools for public health and clinical research, yet they are increasingly vulnerable to sophisticated fraudulent responses, impacting data integrity and quality. Fraudulent responses are submissions that contain false, fabricated, or misleading information provided to misrepresent participation eligibility and that may include automated entries from bots or repeated entries by the same actor [1]. Pinzón et al [2] documented a dramatic erosion of usable data over time, underscoring how AI-enabled fraud and incentive-seeking behavior can overwhelm open links. Similar issues have been reported in nursing and medical research, where 70% to 94% or more of survey responses were identified as fraudulent or automated through bots [3-6]. These findings suggest that traditional safeguards, such as CAPTCHA, are insufficient for data integrity [3,7]. This study’s purpose is to describe the results of implementing a multilayered security approach that integrates automated and human-review mechanisms in a nationwide survey of health care professionals.

## Methods

### Overview

We conducted a cross-sectional online survey of health care professionals across the United States between November 2024 and May 2025. Clinicians aged 18 years or older practicing in the United States who have worked with patients with limited English proficiency were eligible to participate. The survey examined pain-related communication challenges with Spanish-speaking patients with limited English proficiency.

Recruitment occurred through medical professional organization newsletters and websites, health care–focused Facebook and WhatsApp (Meta Platforms Inc) groups, and the authors’ professional Facebook and Instagram (Meta Platforms Inc) pages. No paid advertising was used. We used a 2-tiered process, where posts included a direct link or QR code to the screening survey and then eligible respondents received an individualized link to a Qualtrics (Qualtrics LLC) survey.

Our multilayered security approach combines automated fraud indicators (Table 1) with a human verification process. Some indicator thresholds were adapted from prior literature based on patterns observed in our previous fraud detection work and in the current dataset. Using R software (version 4.5.1; R Foundation for Statistical Computing), we developed a fraud detection workflow that followed these steps: (1) entries were automatically disqualified based on eligibility criteria (eg, non–health care providers) and definitive fraud indicators (eg, non-US submissions), (2) remaining submissions underwent manual review where entries with more than 2 indicators received brief verification of fraudulent status, and (3) entries with 1 or 2 indicators underwent human manual verification, including confirming provider credentials using National Provider Identifier records or state professional licensing board databases. No submission was classified as fraudulent based on a single nondefinitive indicator.

**Table 1.** Comprehensive fraud indicators with definitions [2,8,9].

Indicator name	Definition	Threshold or activity considered fraudulent
Technical indicators		
Qualtrics (Qualtrics LLC) RelevantID fraud score	Qualtrics’ proprietary metric, measures the likelihood of fraudulent response based on multiple factors, such as how respondents interact with the survey and the metadata they generate, to spot signs of fraud or abuse. This tool was discontinued in June 2025.	Score of ≥30
Non-US location	Location of submission identified as outside United States based on IP^a^ address.	Location outside United States
High duplicate score	Qualtrics’s built-in metric, measures similarity between survey responses to detect potential duplicates.	Score of ≥75
Duplicate name	Exact name match in 2 or more responses with different accompanying information.	≥2 occurrences
Duplicate email	Exact email match in 2 or more responses with different accompanying information.	≥2 occurrences
Duplicate IP	IP address that matches in 2 or more responses.	≥2 occurrences
IP block	Submission from known IP blocks associated with VPNs^b^ or proxy services—suspicious when combined with other indicators.	1 or 2 blocks
Disposable email	Sequential submissions from same host or suspicious email domains. Temporary email services act as strong fraud indicators [8].	List of disposable email providers frequently associated with fraudulent activity
Invalid email	Found by format validation.	No @ symbol
Temporal indicators		
Regular waves	Submissions occurring in waves or at regular intervals (eg, 3 responses every 5 min), especially when batches of responses are submitted within 60 seconds at consistent intervals.	≥3 submissions per batch, ≥3 intervals
Rapid submission	Patterns of responses arriving in batches, often dozens per minute, particularly overnight.	≥3 in the same minute
Email pattern indicators		
Many consecutive consonants	Character sequence with 4 or more consecutive consonants. This pattern rarely occurs in authentic email addresses, suggesting random character generation.	≥4 consecutive consonants
Many transitions	Alternating letters and numbers in an email address, which indicates algorithmic construction (eg, a12bcd34e@email.com).	≥2 transitions
Has long number	Addresses with 4 or more digits, which previous work found may indicate automated generation rather than meaningful numbers such as graduation years [9]. Based on patterns observed in our previous work and in the current dataset, we used a threshold of 3 or more digits as a screening indicator requiring further review.	≥3 digits
Low vowel/consonant ratio	A mathematical approach to detect unnatural letter distribution, which emerged from our pattern analysis of confirmed fraudulent addresses.	ratio of ≤0.257
Capitalized names	In the current dataset, structures like “JaneDoe” appeared exclusively in fraudulent responses, while legitimate respondents either used lowercase formatting or included proper punctuation (eg, “Jane.Doe”). This consistent finding likely results from spam generation software attempting to mimic authentic names without understanding professional email conventions [2,8].	JaneDoe format
Long local part	A long email handle; previous work found that no legitimate respondents had email handles exceeding 22 characters [2].	≥18 characters
Starts with number	First character is a number.	Numeric start
No vowels in the email	An extreme deviation from natural language patterns, which emerged from our own analysis as the strongest single indicator of fraudulent generation.	0 vowels
Content indicators		
Honeypot	Interaction with hidden form fields designed to catch automated submission. Survey respondents see a question that says “leave blank” or a variation of it.	Not empty
No contact information	Contact information for subsequent emails is empty.	Missing/empty

a IP: internet protocol.

b VPN: virtual private network.

### Ethical Considerations

This study was approved by the University of Wisconsin-Madison’s institutional review board (ID: 2024‐1314). Informed consent was displayed in the introduction of the survey, and all participants had to agree to participate to continue. Survey responses were stored separately from contact information to ensure privacy and confidentiality. Participants received an electronic gift card of US $15 for completing the survey.

## Results

Our multilayered security approach identified 5846 of 5886 entries (99.32%) as potentially fraudulent, while only 40 were verified to be legitimate. The automated screening phase disqualified 2895 (49.18%) submissions. The remaining 2991 (50.82%) submissions underwent manual review. Of these, 2224 (74.35%) contained 2 or more indicators of fraud and required only brief verification, while 767 (25.64%) required more extensive examination. Table 2 shows that the most frequent fraud signal was the temporal regular waves pattern (n=4447, 75.55%), followed by a high fraud score (n=1614, 27.44%) and duplicate internet protocol (n=1614, 24.88%). Combined attacks were also notable, with the regular wave  + suspicious email pattern being present in 418 (7.1%) responses (Table 2).

**Table 2.** Fraud indicators identified in the survey responses between November 2024 and May 2025 (N=5886).

Indicator name	Count, n (%)
Technical indicators	
High fraud score	1614 (27.44)
Duplicate IP^a^	1464 (24.88)
IP block	860 (14.61)
Non-US location	733 (12.45)
High duplicate score	606 (10.3)
Duplicate name	177 (3.01)
Duplicate email	168 (2.85)
Disposable email	74 (1.26)
Invalid email	5 (0.08)
Temporal indicators	
Regular waves	4447 (75.55)
Rapid submission	1311 (22.27)
Email pattern indicators	
Long number	1261 (21.43)
Many consecutive consonants	935 (15.89)
Capitalized names	589 (10.01)
Low vowel/consonant ratio	302 (5.13)
Long local part	209 (3.55)
No vowels	202 (3.43)
Many transitions	142 (2.41)
Starts with number	21 (0.36)
Content indicators	
No contact information	80 (1.36)
Honeypot	0 (0)
Combined attacks	
Regular wave pattern + suspicious email pattern	418 (7.1)
High fraud score + regular wave pattern	279 (4.74)
Rapid submission + regular wave pattern + suspicious email pattern	279 (4.74)
Rapid submission + regular wave pattern	209 (3.55)
Regular wave pattern + email has 3 or more consecutive digits	201 (3.41)

a IP: internet protocol.

## Discussion

### Principal Findings

This multilayered security approach proved highly effective at identifying submissions with multiple indicators of fraud and distinguishing them from submissions from verified health care professionals through credential verification and manual review. Among individual signals of fraud, the most discriminative was the temporal regular wave pattern. By contrast, the honeypot captured no entries, and submissions with no contact information were rare (1.4%) and nonspecific. This suggests that contemporary threats originating from sophisticated automated systems are capable of avoiding traditional bot-detection mechanisms.

No single indicator was sufficient for adjudication because legitimate circumstances could generate these signals. For example, regular wave patterns may result from social media distribution, duplicate IP addresses may occur when using shared networks, and character-based indicators (eg, a low vowel/consonant ratio) may reflect legitimate naming conventions (eg, Schmidt). Thus, human review is essential to minimize false positives when scaling verification.

Our findings confirm that no single indicator is sufficient; detection improves when multiple indicators are combined [2,8]. Guy et al [10] reported similar results in 2 online studies of marginalized populations, showing that reliance on a single fraud detection approach would have resulted in the majority of bots or fraudulent responses remaining undetected. In our data, even the strongest single signal would have missed nearly one-quarter of fraud if used alone. Multilayered defenses enabled the accurate removal of 16% to 88% of fraudulent entries depending on context; where permissible, personal identifiers would further improve yield [3,7,10,11].

Several limitations should be acknowledged. First, we cannot formally estimate sensitivity, specificity, or overall classification accuracy, including naming traditions, because there is no gold standard to determine each submission’s true status. Second, all recruitment channels used the same screening survey; therefore, we could not compare fraud rates across channels.

As online survey fraud continues to evolve, researchers must remain vigilant and adaptive. The extreme fraud rate documented here may reflect a new reality for online studies, particularly those offering monetary incentives. While resource intensive, our results demonstrate that comprehensive fraud detection can successfully preserve data integrity even in heavily targeted surveys. Our findings also highlight the importance of recruitment strategies. Public social media posts through professional organizations and health care–focused groups may increase survey visibility and simultaneously increase exposure to fraud. Future studies should evaluate whether recruitment through professional organizations’ members-only platforms reduces fraud while maintaining adequate reach.

### Conclusions

This study demonstrates that safeguarding online health survey data requires a comprehensive multitiered framework. As technology evolves, adaptive approaches will be essential to maintain the validity, credibility, and reproducibility of web-based research. Furthermore, we provide practical insights into recruiting health care professionals through professional organizations and public social media channels. Investigators should anticipate recruitment inefficiencies, and they may need to cast a wider recruitment net than anticipated and allocate substantial resources to response verification.

## Abbreviations

IP: internet protocol
VPN: virtual privacy network
